# Reconstruction of facial defects with local flaps – a training model for medical students?

**DOI:** 10.1186/s13005-015-0087-4

**Published:** 2015-09-03

**Authors:** Florian Bauer, Steffen Koerdt, Niklas Rommel, Klaus-Dietrich Wolff, Marco R. Kesting, Jochen Weitz

**Affiliations:** Department of Oral and Maxillofacial Surgery at the Klinikum rechts der Isar, Technische Universität München, Ismaningerstrasse 22, 81675 Munich, Germany

**Keywords:** Teaching, Plastic-reconstructive surgery, facial defects, OMFS

## Abstract

**Introduction:**

The lack of surgeons will be a future major problem in patient care for multifaceted reasons. Niche specialties such as OMFS face an additional drawback because of the need for dual qualification. Special surgical training that gives students the opportunity to gain experience in the techniques of plastic-reconstructive surgery (PRS) has therefore been established to promote interest in OMFS.

**Methods:**

Two hands-on courses with 8 modules of 2 h for 10 students were established. Course modules included surgical techniques of PRS, such as local flaps in a complex facial defect on pig heads, and were supervised by two OMFS surgeons. The identical initial and final tests examined theoretical knowledge and practical skills. Questionnaires concerning basic demographic data, future career goals, and perception of surgical disciplines before and after the completion of the course were handed out.

**Results:**

The 19 participating students (12 female, 7 male; median age 24 ± 2.24) were in their 8.31 ± 1.20 semester. Results of the tests showed improvement in knowledge following the courses (before 52.68 ± 12.64 vs. after 77.89 ± 11.37; *p* < 0.05). Based on the Likert scale, an increase in interest in a career in OFMS was observed (3.90 ± 1.18 vs. 2.72 ± 1.33; *p* < 0.05), but this was not so marked with regard to a career in a surgical discipline in general (1.93 ± 1.30 vs. 1.62 ± 1.19; *p* > 0.05). Perception of OMFS as a surgical discipline changed (3.68 ± 1.09 vs. 1.80 ± 0.64; *p* < 0.05). The following values also changed: students´ perception of PRS in OMFS (14 (74.68 %) vs. 5 (25.32 %); 19 (100 %) vs. 0 (0 %)), evaluation of PRS as a study subject for medical students (7 (36.84 %) vs. 12 (63.16 %); 19 (100 %) vs. 0 (0 %)), and the interest in an OMFS elective subject (6 (31.58 %) vs. 13 (68.42 %); 18 (94.74 %) vs. 1 (5.26 %)) and as a final clinical year subject (4 (21.05 %) vs. 15 (78.95 %); 14 (73.68 %) vs. 5 (26.32 %)).

**Conclusions:**

Hands-on courses with complex facial defects can be used to gain new professionals, even in niche specialties such as OMFS. Moreover, a hands-on course design, including innovative teaching methods and structured objective tests combined with a close student-teacher relationship and motivated instructors, is able to promote complex surgical skills in PRS.

## Introduction

In the past, much effort has been made to interest young medical students in a career in academic surgery [1]. This seems to be especially important, as general interest in a surgical career is declining over time in medical schools [2]. Commonly, interest in surgery as a medical discipline is at a low level amongst medical students and young professionals [3]. Previous studies have shown that teaching itself has a major impact on the awareness of a medical discipline and, consequently, the attitude towards that specialty [4–6]. In course evaluations, in particular, concepts that involve practical tutorials and participation in operative procedures are rated as extremely valuable and account for a positive appreciation of the speciality [5, 7]. Even short workshops that are of only one hour in length and that convey a positive representation of the discipline are significantly able to change attitudes towards and perceptions of surgery [8, 4]. This seems especially true for plastic surgery as a report from the literature by Davis et al. proved [9]. The imminent shortage of surgeons in the near future illustrates the need to motivate young professionals with regard to a career in surgery or a surgically orientated specialty [10]. This is especially true for the surgical field of Oral and Maxillofacial Surgery (OMFS). In many countries, dual degrees from medical and dental school are a prerequisite for the successful completion of residency. The long educational period, which comprises two complete university degree courses, might argue against choosing this subject as a future residency. Indeed, OMFS is a highly diversified specialty, which includes traumatology, cleft-lip-palate surgery, surgical oncology, orthognathic surgery, and especially plastic reconstructive surgery (PRS). However, many students associate only dental implantology with OMFS [11]. The various subspecialties within OMFS are usually affiliated with neighboring disciplines such as Otolaryngology, Pediatrics, and Plastic Surgery. The surgical reconstruction of facial defects following procedures such as ablative cancer surgery involve complex and advanced techniques that can fascinate young medical professionals and medical students alike. Therefore, an innovative curriculum has been developed that is aimed at teaching basic principles of reconstructive facial surgery to medial students in a hands-on course at university. Previous studies by Davis et al. were able to show the impact of a one day training course for a career in plastic surgery in general. 121 participants improved significantly in different key themes [9]. However, especially the reconstruction of facial defects using local flaps in OMFS is challenging for medical students. Therefore, the main goal of the current study has been to investigate the extent to which such complex surgical techniques are suitable for university medial courses and to determine the impact that a hands-on practical course can have on the perception of a surgical subspecialty such as OMFS.

## Methods

### Ethical approval and participants

All courses took place at the Department of Oral and Maxillofacial Surgery at the University of Technology, Munich, Germany. This study followed the Declaration of Helsinki on medical protocol and ethics, and the Institutional Review Board (IRB) of the University of Technology, Munich, Germany approved the study (No. 153/15). All participants were informed extensively and gave consent.

### Course design

**Fig. 1:**
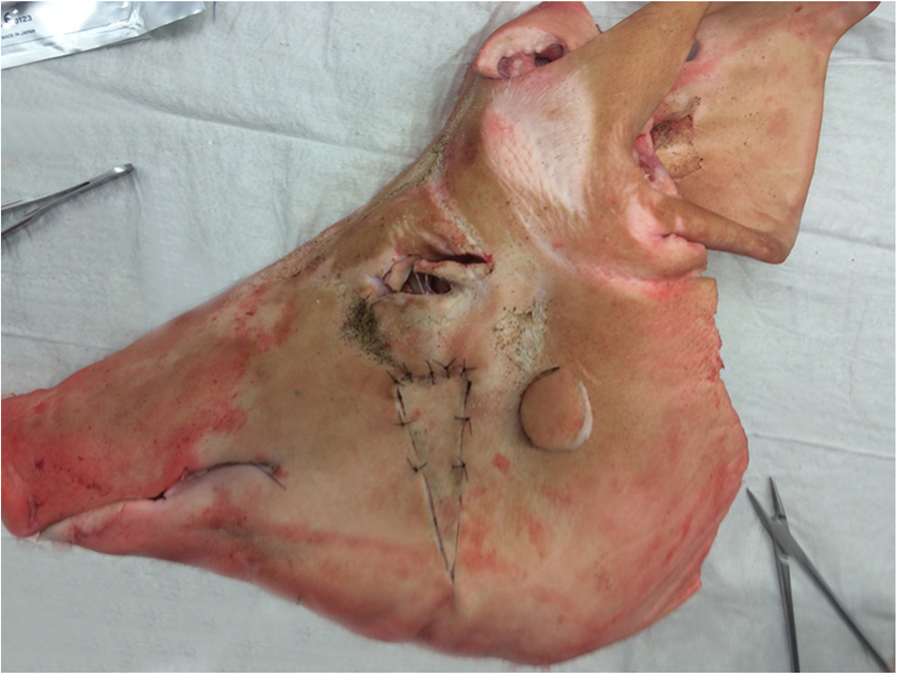
Animal model used in the hands-on course for practicing local skin flaps

Two hands-on courses with 10 students each were undertaken. Each course consisted of an initial written test, eight modules of 2 h, and a final test. Course modules included basic surgical techniques, namely the radial forearm flap (RFF) as an example of microvascular free flaps, neck dissection (ND), full-thickness and split-thickness skin grafts, and local flaps such as the U-flap, kite-flap, rotation-flap, biloped-flap, and z-plasty. Each module was supervised by two OMFS surgeons. This enabled a close 1:5 student:teacher ratio. The identical first and final tests examined theoretical knowledge, which was available for self-instruction on a university-owned online platform. Practical skills were tested by using the Direct Observation of Procedural Skills (DOPS) method. Theoretical modules on RFF and ND were taught by using specially designed anatomical models [12]. Practical course modules consisted of a short theoretical introduction followed by practical tutorials with animal cadaver models, as shown in Fig. 1. Cadaver models were purchased from a local slaughterhouse. Ethical approval for use in medical education was obtained from the local IRB (No. 153/15).

Additionally, access to an online video tutorial was offered for further self-instruction. In the context of the tests, the participants were asked to answer questionnaires on various aspects concerning basic demographic data, future career goals, and perception of surgical disciplines before and after completion of the course (Table 1).

**Table 1:** Questionaire used for course evaluation

Item	Scale
Basic demographic data	
Age	
Semester	
Gender	
Possible career in surgery	Likert (1 = strongly agree to 5 = strongly disagree)
Possible career in OMFS	Likert (1 = strongly agree to 5 = strongly disagree)
Because of dual degree^1^	Yes/No
Because of speciality itself^1^	Yes/No
Perception of OMFS	Likert (1 = very good to 5 = very bad)
Is PRS part of OMFS	Yes/No
Are PRS techniques lernable for student	Yes/No
Interest in further OMFS courses	Yes/No
Interest in an externship in OMFS	Yes/No
Interest in OMFS as part of final year	Yes/No

OMFS: Oral and Maxillofacial Surgery; PRS: Plastic-Reconstructive Surgery

^1^Only had to be answered, if answer to previous question was 4 or 5 on the Likert scale

### Statistical analysis

All data were analyzed by using IMB®SPSS® for Mac (version 22.0; IMB Corp., USA). Means and standard deviation (SD) were calculated, and tests of significance were performed. For normally distributed values, the *t*-test was performed. For values not normally distributed, the Mann–Whitney test was used. Statistical significance was defined as α = 0.05. All *p*-values are local and given as two-tailed.

## Results

A total of 19 students participated in the course and complete the evaluations. Students (12 female, 7 male; median age 24 ± 2.24 years) were in their 8.31 ± 1.20 semester in medical school at the University of Technology, Munich, Germany.

**Fig. 2:**
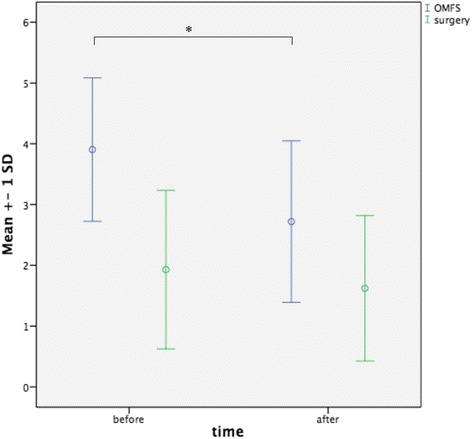
Graphical illustration of students´ perception of OMFS and surgery in general, before and after completion of the course. OMFS: Oral and Maxillofacial Surgery; SD: Standard Deviation; * = *p* < 0.05

**Fig. 3:**
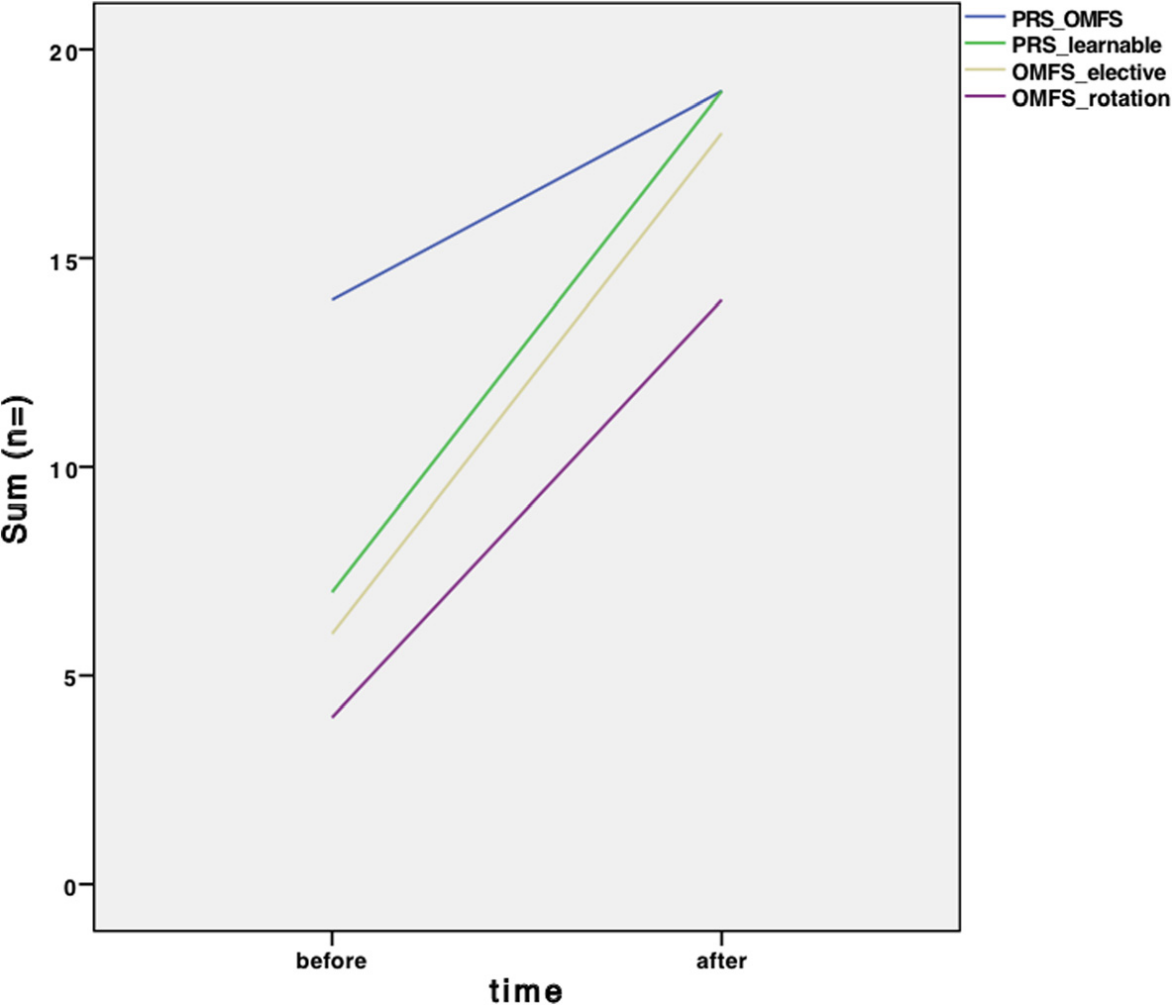
Graphical illustration of the number of students rating PRS as a part of OMFS, the ability to learn PRS techniques in a hands-on course for students, and their interest in elective and final year rotational subjects in OMFS, before and after completion of the course. OMFS: Oral and Maxillofacial Surgery; PRS: Plastic-Reconstructive Surgery

Based on the Likert scale, a statistically significant increase in students who were interested in a career in OFMS could be observed after completion of the course (3.90 ± 1.18 vs. 2.72 ± 1.33; *p* < 0.05; Fig. 2). A smaller increase could be observed concerning a career in a surgical discipline in general (1.93 ± 1.30 vs. 1.62 ± 1.19; *p* > 0.05; Fig. 2). The perception of OMFS as a surgical discipline also changed before and after completion of the course (before: 3.68 ± 1.09 vs. After: 1.80 ± 0.64; *p* < 0.05). Figure 3 visualizes the students´ perception of PRS in OMFS (before: 14 (74.68 %) vs. 5 (25.32 %); after: 19 (100 %) vs. 0 (0 %)), the evaluation of PRS as a subject in teaching for medical students (before: 7 (36.84 %) vs. 12 (63.16 %); after: 19 (100 %) vs. 0 (0 %)), and the interest in an OMFS elective subject (before: 6 (31.58 %) vs. 13 (68.42 %); after: 18 (94.74 %) vs. 1 (5.26 %)) and rotation during the final year at medical school (before: 4 (21.05 %) vs. 15 (78.95 %); after: 14 (73.68 %) vs. 5 (26.32 %)). Results of the tests also showed statistically significant improvement in the knowledge of the students (52.68 ± 12.64 vs. 77.89 ± 11.37; *p* < 0.05).

**Fig. 4:**
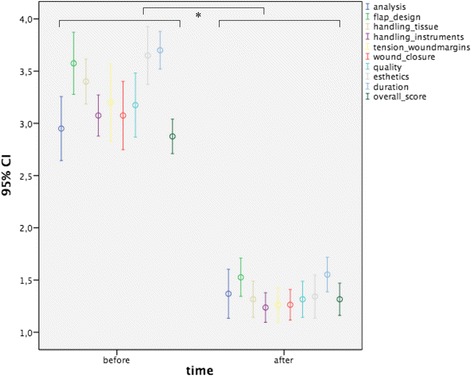
Graphic illustration of the results of the Direct Observation of Procedural Skills (DOPS) method in categories (i) clinical analysis, (ii) flap design used for closure of the defect, (iii) handling of the tissues, (iv) handling of the surgical instruments, (v) increased tension on the wound margins, (vi) sufficiency of wound closure, (vii) overall quality, (viii) overall esthetics, (ix) duration of surgical procedure, and (x) overall score before and after completion of the course; values are given as means with error bars indicating the 96 % confidence intervals (CI); * = all *p* < 0.05

Results of the final DOPS exam are displayed in Fig. 4 (all *p* < 0.05).

## Discussion

In addition to patient care and research, the teaching and education of students are two of the core responsibilities in an academic medical setting. However, especially in surgery, the duties and tasks necessary in every-day patient care often collide with aims in research and teaching. Therefore, the evaluation and analysis of current concepts in teaching are of great importance [13]. The attractiveness of surgery has to be improved as a potential discipline for medical students, as a shortage of surgeons is imminent in the near future. Reasons for this are manifold. Surgical departments face new laws on working hours, an increased demand for a balanced work-life ratio, but also rising expectations for the individual academic surgeon [14]. Only about 9 % of all medical school graduates pursue a career in surgery. During medical school, potential interest in surgery can even decrease over time. Overall, more males than females are interested in the surgical field [10], despite the percentage of female medical students having continuously increased within the last few years [15]. Of all medical students, 60 % are female, whereas only 33 % of young female professionals start a career in surgery [16]. What are the reasons for this development Other studies have been able to show that an early connection with a specialty plays a major role in decision-making by young professionals. Therefore, the satisfaction that results from working in this specialty has to be communicated to students early in their studies. Such emotional aspects can convince young professionals to chose a career in this discipline, despite all other cofactors [17]. Positive experiences and inspiring teachers are able to persuade students to become surgeons [18]. The course presented in this current study with 63 % female participation supports these ideas.

What is essential for a successful educative concept in surgery other workers have emphasized that the establishment of hand-on courses in combination with training courses has a positive effect on education [13]. In particular, the teaching of small groups in surgical courses is evaluated as significantly better than that in large groups. Furthermore, continuity and didactical training of the teaching assistants is important in a successful course design [1]. Our study group has been able to show that a structured hands-on course significantly improves the surgical skills of medical students and their self-assessment of these skills [5].

All participants in the current course were in the 7^th^ to 10^th^ semester in medical school. We assumed inhomogeneous previous knowledge and skills. An initial test checked the students´ level according to the Kirkpatrik Level II “Learning”. The results showed a certain basic theoretical knowledge, but students only possessed rudimentary practical surgical skills, including basic surgical skills. Is the teaching of complex surgical skills such as those required in PRS suitable for student courses, when a lack in basic surgical skills exists Challenges with surgical problems have been shown to represent positive factors in career decision-making of medical graduates [3]. Even the complex surgical techniques needed in PRS, such as the RFF, are suitable for student education purposes [12].

Initially, basic surgical techniques were taught in a two-hour module. Further modules consisted of advanced surgical skills in PRS. Wanzel et al. have shown that a one-on-one hands-on training of just 5 min significantly improves surgical skills of residents in local skin flaps in an animal model [19]. Other studies on local flap techniques in plastic reconstructive surgery described teaching options using foam models for example [20–22].

The results of the final test and the evaluation verify that PRS techniques are suitable for medical student education and can be conveyed in a hands-on course design as described in the current study. This course has been developed and carried out in a department specializing in OMFS. OMFS is a highly diverse surgical subspecialty that involves traumatology, cleft-lip-palate surgery, surgical oncology, orthognathic surgery, and especially PRS. In many countries, dual degrees from a medical and dental school are a prerequisite for the successful completion of residency; this results in a long training period. The combination of a professional and family life is particularly challenging in surgery. This is certainly an aspect that has a major influence on young female and increasingly also on young male professionals when making a decision regarding their career [23]. A variety of important factors influences graduates to chose a career in surgery. These include role models and career chances. Other factors such as lifestyle, long working hours, and the long period of study are rated detrimentally [24]. Results from the current study confirm these findings. However, students had a significantly improved perception of OMFS after completion of the course and could even imagine choosing OMFS as an elective or rotational subject within their final year at medical school. Nevertheless, a certain selection bias certainly has to be kept in mind, as participation in this course was by choice. A fundamental interest in surgery presumably previously existed in this group. The finding that significantly more students were interested in the elective or rotational study of OMFS after completion of this course suggests that an interest in a surgical career can be fostered throughout medical studies. In particular, elective and rotational subjects can improve an interest in surgery significantly [25]. Participation in the operating theater, positive interactions with staff, and integration into a team are experiences that, during an elective study period, can convince students to chose a certain discipline. The high work load and long working hours remain as negative experiences during such an elective subject [26]. Final year rotational subjects, on the other hand, have a dramatic influence on future career development [27]. Therefore, well-structured final year rotational and elective subjects are essential [28].

## Conclusions

Hands-on courses dealing with complex facial defects as presented in this study can gain the interest of young professionals, even for niche specialties such as OMFS. Moreover, a hands-on course design, including innovative teaching methods and structured and objective tests combined with a close student-teacher relationship and motivated instructors, is able to demonstrate even complex surgical skills in PRS. Further studies are necessary to investigate the long-term results as to whether these students did indeed choose OMFS as their future career, whether this kind of training had a positive influence, and the nature of its effects.
